# Small RNAs from the wheat stripe rust fungus (*Puccinia striiformis* f.sp. *tritici*)

**DOI:** 10.1186/s12864-015-1895-4

**Published:** 2015-09-21

**Authors:** Nicholas A. Mueth, Sowmya R. Ramachandran, Scot H. Hulbert

**Affiliations:** Molecular Plant Sciences, Washington State University, Pullman, WA USA; Plant Pathology, Washington State University, Pullman, WA USA

**Keywords:** *Puccinia*, Pathogen, Fungus, Stripe rust, Wheat, Small RNA, miRNA

## Abstract

**Background:**

Wheat stripe rust, caused by *Puccinia striiformis* f. sp. *tritici*, is a costly global disease that burdens farmers with yield loss and high fungicide expenses. This sophisticated biotrophic parasite infiltrates wheat leaves and develops infection structures inside host cells, appropriating nutrients while suppressing the plant defense response. Development in most eukaryotes is regulated by small RNA molecules, and the success of host-induced gene silencing technology in *Puccinia spp*. implies the existence of a functional RNAi system. However, some fungi lack this capability, and small RNAs have not yet been reported in rust fungi. The objective of this study was to determine whether *P. striiformis* carries an endogenous small RNA repertoire.

**Results:**

We extracted small RNA from rust-infected wheat flag leaves and performed high-throughput sequencing. Two wheat cultivars were analyzed: one is susceptible; the other displays partial high-temperature adult plant resistance. Fungal-specific reads were identified by mapping to the *P. striiformis* draft genome and removing reads present in uninfected control libraries. Sequencing and bioinformatics results were verified by RT-PCR. Like other RNAi-equipped fungi, *P. striiformis* produces large numbers of 20–22 nt sequences with a preference for uracil at the 5′ position. Precise post-transcriptional processing and high accumulation of specific sRNA sequences were observed. Some predicted sRNA precursors possess a microRNA-like stem-loop secondary structure; others originate from much longer inverted repeats containing gene sequences. Finally, sRNA-target prediction algorithms were used to obtain a list of putative gene targets in both organisms. Predicted fungal target genes were enriched for kinases and small secreted proteins, while the list of wheat targets included homologs of known plant resistance genes.

**Conclusions:**

This work provides an inventory of small RNAs endogenous to an important plant pathogen, enabling further exploration of gene regulation on both sides of the host/parasite interaction. We conclude that small RNAs are likely to play a role in regulating the complex developmental processes involved in stripe rust pathogenicity.

**Electronic supplementary material:**

The online version of this article (doi:10.1186/s12864-015-1895-4) contains supplementary material, which is available to authorized users.

## Background

*Puccinia striiformis* f. sp. *tritici* (*Pst*) is a Basidiomycete fungus that causes wheat stripe rust disease. Repeated outbreaks in Northern Africa, the Middle East, and Central Asia have contributed to economic hardship and food insecurity in these regions [1]. Stripe rust is also an important cereal disease throughout North America [2]. Transnational and even transcontinental disease proliferation occurs when winds lift rust spores from infected fields into long-distance air currents [3]. Although stripe rust has historically affected cool growing regions, the worst recent outbreaks were caused by strains with an unprecedented tolerance for warmer climates [4].

*P. striiformis* is an obligate biotroph; the fungus produces specialized infection structures called haustoria inside living host cells [5]. The haustorium is the site of nutrient uptake, and also the site for delivery of compounds that influence virulence, known as effectors [6]. In other biotrophic fungi, effector proteins block induction of the plant resistance response, or protect fungal cells from its effects [7]. In turn, plants have evolved resistance proteins to detect the presence of these effectors, leading to effector-triggered immunity [8]. Using the latest *Pst* draft genome and transcriptome, genes coding for proteins with effector-like amino acid sequences were identified for further analysis [9, 10].

The complex two-way interaction between pathogens and their hosts can be partly decoded via patterns of gene expression and regulation. Dual RNA-sequencing of both pathogen and host is an elegant means to explore both sides of this interaction [11, 12]. The full picture of gene regulation during infection includes not only protein-coding genes, but noncoding RNAs as well. Small RNAs (sRNAs) are short noncoding RNA molecules that regulate gene expression in many plant life processes, including developmental timing [13], meristem maintenance [14], and response to pathogens [15]. Small RNAs fall into two main classes: small interfering RNA (siRNA), which originates from a heteroduplex of two distinct RNA molecules, and microRNA (miRNA), which is transcribed from a single-stranded precursor with self-complementarity [16]. Plant miRNAs contribute to resistance by controlling the induction of defense-related genes via post-transcriptional gene silencing (PTGS) [17, 18].

Small RNA from many fungal species have been surveyed since the first discovery of RNAi in *Neurospora* [19]. Several species, including *Saccharomyces cerevisiae* and the plant pathogen *Ustilago maydis*, were found to have lost their RNAi capability [20–22]. However, many fungi and oomycetes, including pathogenic ones, carry functional small RNAs [23–25]. Small interfering RNAs (siRNAs) from the necrotrophic fungus *Botrytis cineria* function as virulence factors by silencing plant defense genes [26, 27]. A biotroph such as *Pst*, which maintains an intimate relationship with its host both physically and evolutionarily, might be particularly adapted to employ sRNA-based effectors [28].

Fundamental research on post-transcriptional gene silencing in parasitic fungi has led to a tantalizing prospect for molecular genetic control of pathogen virulence via host-induced gene silencing (HIGS) [29]. HIGS works by expressing antisense RNA interference (RNAi) constructs in host cells, which often results in silencing of complementary genes in the pathogen [30, 31]. Reduced virulence phenotypes were obtained in the leaf rust fungus *P. triticina* and stem rust fungus *P. graminis* using this technology [32, 33]. However, no study to our knowledge has surveyed the small RNA population of any *Puccinia* species, whether endogenous or HIGS-induced. Much remains unknown about the fungal gene silencing machinery in general; some evidence suggests there are sRNA biogenesis pathways found only in fungi [34]. The goal of this study was to help fill these gaps by describing the small RNA repertoire of *Pst*.

Unlike many other pathogenic fungi, such as *Magnaporthe* or *Botrytis*, it is currently not feasible to raise axenic cultures of *P. striiformis* in the laboratory. Thus, obtaining samples during development must involve extracting RNA from infected plant tissue, and then removing contaminating wheat sequences [35]. In this work, we performed small RNA-sequencing on infected wheat, then used bioinformatic and molecular techniques to identify fungal-specific sRNA reads. These sequences were shown to share structural properties with previously-described fungal sRNA libraries, including microRNA-like sequences. This study also contributes a large list of predicted sRNA-target pairs, and identifies specific biological processes that may be regulated by PTGS.

## Results and discussion

Two soft white spring wheat cultivars, ‘Penawawa’ and ‘Louise’, were chosen as host plants. Penawawa is susceptible to strain PST-100, whereas Louise possesses partial high temperature adult plant (HTAP) resistance, largely controlled by a locus on chromosome 2BS [36]. We speculated that the partially resistant Louise would provide a challenging host environment for the pathogen, yet still enable significant accumulation of fungal biomass (hence RNA). Analysis of various cultivars could reveal differences in the fungal sRNA repertoire between compatible and partially incompatible interactions.

Fully-emerged flag leaves on 6 week-old wheat plants were inoculated with either PST-100 spores mixed with talcum powder, or mock-inoculated with talcum powder only. There were 4 treatment groups: Infected Penawawa (IP), Infected Louise (IL), Uninfected Penawawa (UP), and Uninfected Louise (UL). Three biological replicates (individual plants) were in each treatment group; there were 12 samples total. Flag leaf tissue was collected for RNA extraction at four days post-inoculation (dpi). This time point corresponds to a high rate of haustorium growth [37], and falls within a critical period in the development of biotrophic infection. Disease symptoms were not visible to the naked eye at this stage; flag leaves from all treatments appeared very similar. By 14 dpi, uredinia had developed on infected plants from both cultivars, although Louise plants showed less disease severity than Penawawa. No mock-inoculated plants ever developed pustules. Total RNA was extracted from each sample and divided into two sub-samples. One half remained as total RNA for RT-PCR analyses. The other half was size-selected for short RNAs (<200 nt), ligated to adapters, reverse transcribed, and sequenced via the Ion Torrent platform.

### *P. striiformis* expresses RNA interference genes during infection

Prior genome analysis of *Pst* race 130 predicted several genes required for small RNA-mediated gene silencing, including Dicer-like (RNAse III) and Argonaute genes [9]. BLAST searches confirmed that genes with high sequence similarity to Dicer (*PSTG_15713*) and Argonaute (*PSTG_06326*) are also present in a different draft genome: PST-78 [38]. Also, at least two hypothetical proteins (*PSTG_03098.1*, *PSTG_15184.1*) are highly similar to an RNA-dependent RNA polymerase necessary for the quelling of transgenes in *Neurospora crassa* (*QDE-1*).

**Fig. 1:**
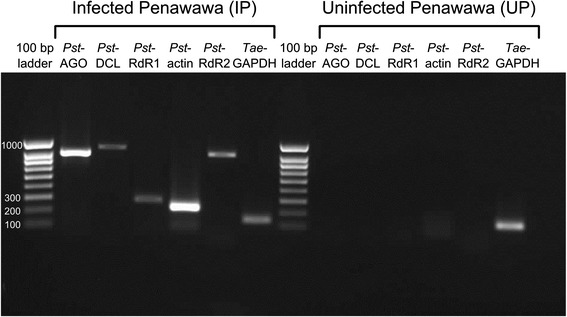
RT-PCR to detect *P. striiformis* RNA interference genes in infected wheat tissue. Stripe rust transcripts similar in sequence to Argonaute (*Pst-AGO*), Dicer-like (*Pst-DCL*), and RNA-dependent RNA polymerase (*Pst-RdR1*, *Pst*-*RdR2*) were amplified via RT-PCR. *Pst-actin* and wheat *GAPDH* were used as controls. Results for Infected Penawawa (left), and Uninfected Penawawa (right)

To determine whether these genes are expressed during stripe rust infection, reverse transcription followed by PCR (RT-PCR) was performed on the total RNA extracts. Fragments of all four genes were successfully amplified from infected Penawawa plants, and were not observed in the uninfected Penawawa control (Fig. 1). The experiment was repeated for all three replicates in each treatment with similar results. PCR products were sequenced to confirm that they match the correct fungal gene sequences.

### Sequencing results, mapping, and analysis

Small RNA sequencing generated over 50 million total reads between 18–40 nt in length (Table 1a). Not counting redundant reads, there were 3–5 million different sequences in each treatment, nearly 16 million in all (Table 1b). Similar sequencing depth was achieved with uninfected plants of both partially resistant (Louise) and susceptible (Penawawa) wheat varieties.

**Table 1:** Results of small RNA sequencing. Counts of: total reads from 18–40 nt; reads mapping to the *P. striiformis* draft genome; reads remaining after uninfected library subtraction; and reads remaining after removing reads matching wheat miRNA and protein-coding genes

Treatment	IL	IP	UL	UP	Total
1a.					
Total reads	12,406,438	10,462,148	16,947,323	16,581,382	56,397,291
Reads mapping to *Pst* genome	190,677	176,960	196,183	185,452	749,272
Reads mapping to *Pst* genome (%)	1.5	1.7	1.2	1.1	
After subtracting uninfected	50,909	38,362	--	--	89,271
After filtering	45,754	33,805	--	--	79,559
1b.					
Non-redundant sequences	3,539,878	2,921,622	4,982,417	4,534,040	15,977,957
Sequences mapping to *Pst* genome	44,668	39,717	29,913	30,655	144,953
Sequences mapping to *Pst* genome (%)	1.3	1.4	0.6	0.7	
After subtracting uninfected	24,107	18,843	--	--	42,950
After filtering	20,269	15,490	--	--	35,759

Treatment-level counts are the sum of three replicates. 1a. Total reads, including redundant reads. 1b. Nonredundant (unique) sequences only

*IL* Infected Louise, *IP* Infected Penawawa, *UL* Uninfected Louise, *UP* Uninfected Penawawa

To help identify a set of fungal-specific sRNA reads present in infected libraries, all reads were first mapped to the *P. striiformis* PST-78 draft genome. Approximately 1.3 % of all nonredundant sequences in the infected Louise treatment mapped with zero mismatches to the *Pst* genome (Table 1b). However, 0.6 % of sequences from *uninfected* Louise also mapped to the fungal genome. Overlap between host and pathogen noncoding RNA has also been observed in the rice blast fungus *Magnaporthe oryzae* [18]. Small fragments from structural RNA families that are conserved among eukaryotes, as well as transcription from low-complexity regions, can cause natural overlap between infected and uninfected sRNA libraries. Since some wheat-based reads may have mapped to the fungal genome by chance, a library subtraction technique was used, taking advantage of the uninfected controls (illustrated in Additional file 1). Sequences from a given infected variety were only considered likely to be of fungal origin if they: 1) perfectly matched the *Pst* genome, and 2) were never found in the corresponding uninfected replicates of that variety. For example, 50,909 mapped reads were found in Infected Louise, but never in Uninfected Louise (Table 1a).

To further increase stringency, reads matching wheat miRBase entries were filtered out [39]. Finally, reads with a perfect match to the Washington Wheat Transcriptome, containing 190,000 unique gene sequences [40], were removed. The rationale for doing so was to discard any short fragments of wheat genes that are only transcribed during stripe rust infection (and would therefore remain after subtracting the uninfected control library). On the other hand, such a filter might remove important fungal sRNAs that are perfectly antisense to wheat genes. Therefore, BLAST results were limited to only remove hits in the sense (protein-coding) orientation. This strategy successfully removed reads that ambiguously matched the known transcriptome of both organisms. While some legitimate fungal sequences may have been lost in this process, thousands remained after filtering (Table 1a, b).

### Confirmation of sequencing results by RT-PCR

**Fig. 2:**
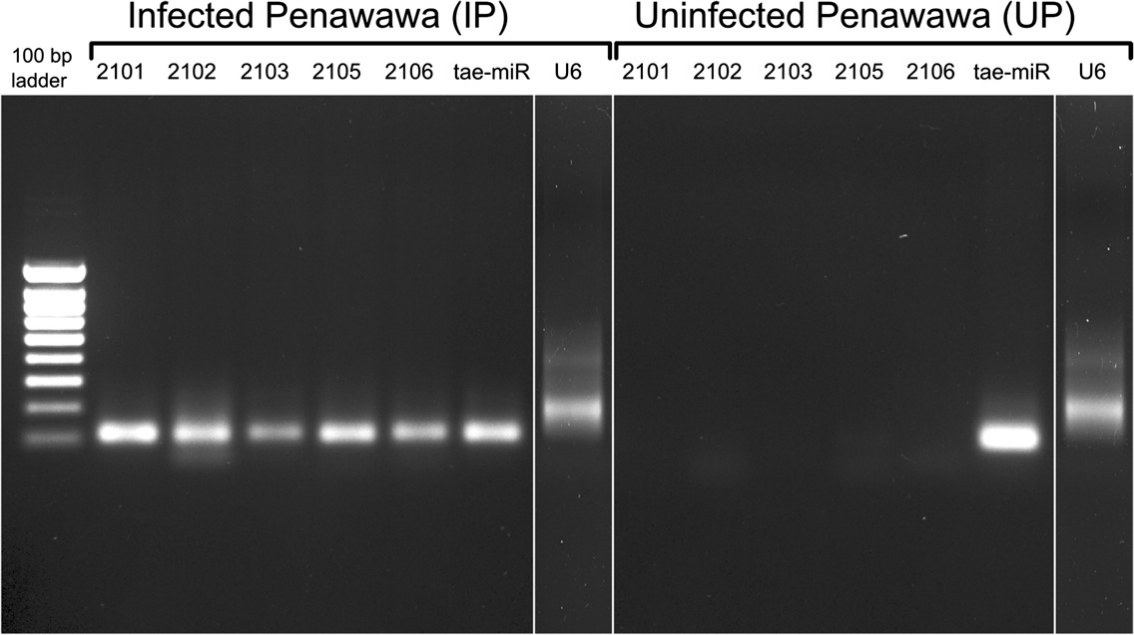
RT-PCR to detect *Pst-*sRNAs from infected wheat tissue. Five *Pst*-sRNAs (named IP_2101, IP_2102…, IP_2106) with mean abundance >10 reads/library were amplified via RT-PCR. A wheat miRNA (tae-miR9772) and U6 snRNA were used as positive controls. For clarity, U6 lanes were re-arranged to be placed next to each treatment. Results for Infected Penawawa (left) and Uninfected Penawawa (right)

An RT-PCR method optimized for small RNA was used to check the results of RNA-seq [41]. Five 21-nt sequences attributed to *P. striiformis* using the mapping, subtraction, and filtering approach were chosen. Amplification was observed in infected tissue samples, but not in the uninfected controls (Fig. 2). As expected, a known wheat miRNA and a small nuclear RNA amplified from both infected and uninfected samples. The experiment was repeated for all three replicates of both wheat varieties with similar results. Therefore, laboratory results support the assertion that sRNA reads bioinformatically assigned to *Pst* do indeed originate in the fungus, and are not contamination from wheat.

### Characteristics of *Pst*-sRNA sequences

**Fig. 3:**
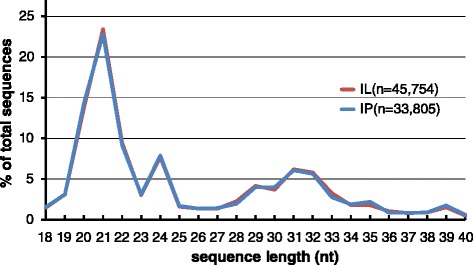
*Pst*-sRNA length distribution. Line chart displaying the relative abundance of three length classes of stripe rust sRNA: 20–22 nt, 23–25 nt, and ≥26 nt. IL = Infected Louise; IP = Infected Penawawa

We hypothesized that *P. striiformis* small RNAs (*Pst*-sRNAs) are processed in a Dicer-dependent manner. Under the null hypothesis, nonspecific RNA degradation would be the primary source of sRNA reads, and particular sequences with fixed lengths would not accumulate. However, the size distribution clearly deviated from the random or flat distribution expected in the absence of sRNA biogenesis (Fig. 3). A pronounced peak at 20–22 nt and a smaller peak at 24 nt are consistent with functional sRNA libraries from diverse eukaryotes. This distribution differs from sRNA size distributions from RNAi-deficient fungi like *Saccharomyces cerevisiae* [22]. There was also a broad peak of sequences 26 nt in length or greater. Long sRNAs have occasionally been observed in previous small RNA studies in fungi [42].

**Fig. 4:**
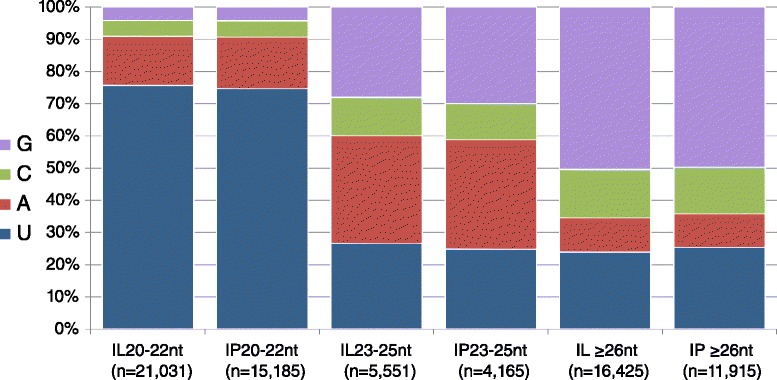
Relative nucleotide frequency of the 5′ end of *Pst-*sRNAs. Stacked bar charts displaying the relative frequency of each nucleotide in the first position of three length classes: 20–22 nt, 23–25 nt, and ≥26 nt. Length classes were chosen empirically from the three prominent peaks shown in Fig. 3. Bars designated ‘IL’ correspond to sRNAs from the Infected Louise library, and ‘IP’ to the Infected Penawawa library. All bars add to 100 %

From the three prominent peaks in the distribution, we pooled *Pst-*sRNA sequences into three size classes: 20–22 nt, 23–25 nt, and ≥26 nt, then calculated the relative frequency of each nucleotide at the 5′ (first) position. A majority (75 %) of 20–22 nt *Pst*-sRNA sequences began with uracil, whereas guanine and cytosine were suppressed (Fig. 4). For consistently-expressed sequences (at least one read in all infected replicates), the proportion of 5′U rose to 85 %. This result closely matches the small RNA population of *Neurospora crassa*, which reported 82 % uracil at the 5′ end [42]. As with the length distribution, this 5′ nucleotide preference was not observed in the RNAi-deficient *S. cerevisiae* [22]. Meanwhile, the 23–25 nt and ≥26 nt *Pst-*sRNA sequences showed moderate biases for adenine and guanine, respectively (Fig. 4).

Many *Pst*-sRNA sequences accumulated dozens or hundreds of times in each library (Additional file 2). However, sRNA sequences >26 nt also showed far more length polymorphism than the shorter ones, with multiple length variants of otherwise identical sequences. This suggests the absence of precise processing for the longer length classes. Taken together, the size distribution, 5′ nucleotide bias, and expression levels of 20–22 nt *Pst*-sRNA sequences are consistent with the idea that they are transcribed and processed in a specific manner. In contrast, the same characteristics did not hold for longer sRNAs. These results are expected if a Dicer-dependent RNA biogenesis pathway is active in this organism.

The size distribution and 5′ nucleotide usage of *Pst-*sRNAs from the two infected cultivars were virtually identical (Figs. 3 and 4). The set of sequences found in the two infected cultivars were similar, but not identical. All 20–22 nt *Pst-*sRNA sequences with moderately high expression levels (>30 total reads) were found in both IP and IL. After Empirical Analysis of Differential Gene Expression (EDGE) at an FDR-adjusted p-value of 0.05, no sRNA sequences in this length class were found to be differentially expressed. On the other hand, some longer sRNAs (≥30 nt in length) were both abundant and unique to either infected Louise or infected Penawawa (Additional file 2). All of these longer sequences had less-abundant but nearly identical length variants in both infected libraries. Despite the HTAP resistance present in ‘Louise’, we did not observe large differences in the fungal sRNA populations between the two infected cultivars.

### *P. striiformis* produces microRNA-like sequences

MicroRNAs originate from a single-stranded RNA precursor, which folds into a characteristic stem-loop secondary structure when transcribed. Dicer activity then cleaves the precursor into a miRNA/miRNA* duplex with two-nucleotide 3′ overhangs [16]. Some fungi, including *Neurospora crassa* and *Penicillium marneffei*, are known to produce microRNA-like RNAs (milRNA) [42, 43]. To investigate whether *Pst* carries milRNA, we used the ShortStack software package [44], which has been used to discover miRNA loci in diverse eukaryotes. The main ShortStack program first searches a mapping file for clusters of overlapping sRNA reads. A second program, Maple, analyzes the genomic region flanking such clusters for evidence of a stem-loop precursor capable of producing miRNAs. Finally, a score is calculated indicating the likelihood that a given locus is a genuine miRNA locus.

**Fig. 5:**
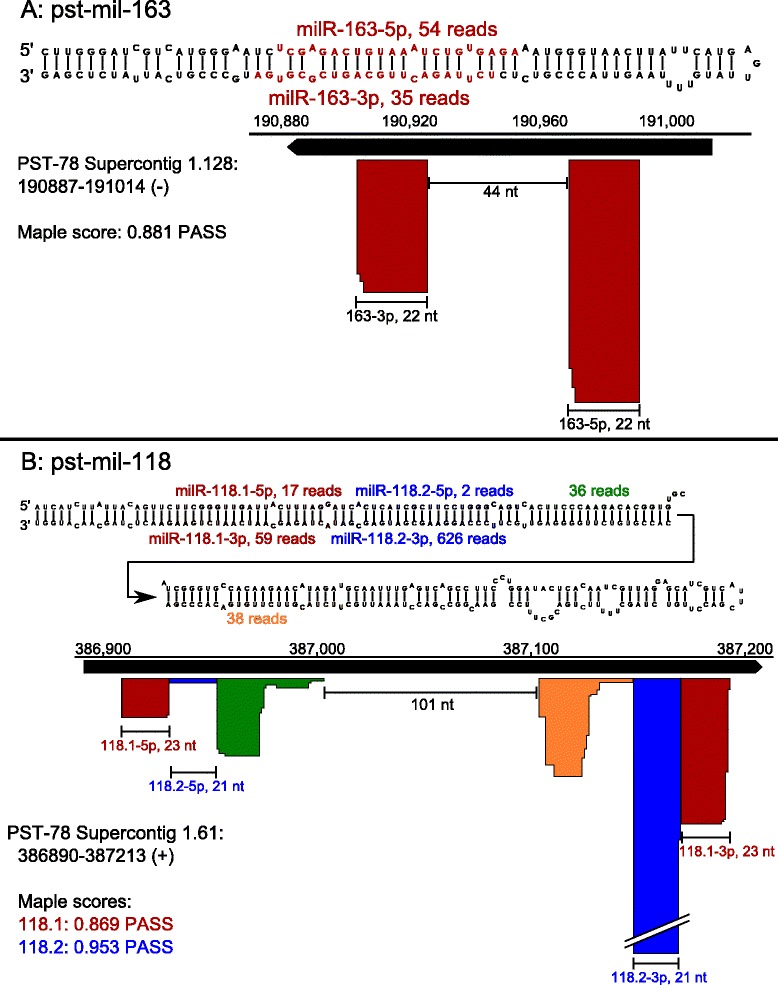
microRNA-like loci in *P. striiformis*. Predicted secondary structure of two miRNA-like (mil-RNA) precursors and distribution of reads along the genomic region. Colored boxes indicate the depth of mapped reads corresponding to specific sRNA sequences (colored text). **a**. Predicted secondary structure of pst-mil-163. **b**. Predicted secondary structure of pst-mil-118. Arrow indicates the continuation of a long stem-loop precursor. Two miRNA/miRNA* duplexes met the criteria for precise excision from the precursor (red and blue); the others did not (green and orange)

ShortStack identified a 128 bp region on Supercontig 1.128, between two predicted genes (*PSTG_12821* and *PSTG_12822*) and near, but not overlapping, multiple Harbinger and Copia transposable elements. All reads in this region mapped to a single strand of the DNA sequence. If transcribed, this region would assume a stem-loop secondary structure with two clusters of 22 nt mapped sRNA reads (Fig. 5a). The two sequences form a duplex on opposing arms of the hairpin with the expected two-nucleotide 3′ overhangs. In addition to being similar to *Neurospora* milRNA [42], this pattern meets the primary criterion used in the annotation of plant microRNA: “precise excision from a stem-loop precursor” [45].

A minor criterion for miRNA annotation is conservation among related species, but BLAST searches with the pst-mil-163 precursor sequence against the *P. graminis* and *P. triticina* genomes returned no significant alignments. Within the PST-78 draft genome, five regions with >75 % sequence identity were identified; all were on the same supercontig (1.128). However, none of these additional loci had the depth or pattern of mapped sRNA reads that would indicate multiple members of the pst-mil-163 family.

Another locus identified by Maple as miRNA-like is located on Supercontig 1.61. Interestingly, two adjacent pairs of milRNA sequences were predicted on a single long precursor (Fig. 5b). Pst-milR-118.2-3p is a 21 nt sequence with over 600 reads in the total infected library; its miRNA* sequence, pst-milR-118.2-5p, was also present. Pst-milR-118.1 has a precise 5′ start site and varies in length by 1–2 nt at the 3′ end. A relatively long loop region (>100 nt) lies between the two main clusters. Unlike the two milRNA duplexes, reads within the loop region varied in both length and 5′ start site, and did not directly oppose one another on the precursor sequence. Multiple distinct miRNAs from a single precursor were previously described in plants [46, 47]. It will be useful to search for similar loci in related species.

### *Pst*-sRNA loci overlap with functional genome annotations

ShortStack outputs an annotation file with the genomic coordinates of all clusters of mapped reads above a user-defined threshold. Using the default threshold of 20 overlapping reads, 138 clusters were detected in IL and 112 clusters in IP (Table 2). Additionally, RNA-seq reads from all infected replicates (both IL and IP) were combined to create a “pooled infected” library, and ShortStack analysis was repeated to obtain 187 total clusters meeting the minimum depth threshold (Table 2). Virtually all clusters in that pooled library were composed of reads from both IP and IL; there were only a few small clusters unique to one infected treatment or the other. Thus, the two different host cultivars did not appear to induce any obvious presence/absence changes in fungal sRNA production. ShortStack clusters were dispersed across 80 different genomic supercontigs. Since the current draft genome contains 9,700 unordered supercontigs, and chromosomal information is lacking, it is currently impossible to draw any conclusions about the genome-wide distribution of *Pst-*sRNA loci.

**Table 2:** *Pst*-sRNA loci that overlap with stripe rust genome annotations

	Infected Louise		Infected Penawawa		Pooled IL&IP	
	Loci	Reads	Loci	Reads	Loci	Reads
*Pst* genes	21	3,627	21	2,183	33	7,694
tRNA	68	12,318	56	8,667	89	21,460
RepBase	15	1,161	13	920	18	2,456
Rfam	2	219	2	180	2	404
No annotation	32	4,741	20	2,889	45	8,798
Total	138	22,066	112	14,839	187	40,812

Previous fungal studies found that many sRNA loci overlap with features such as genes, transposons and tRNAs [25, 26]. We compared the locations of sRNA loci identified by ShortStack with various genomic annotations, and counted the number of overlapping features. Approximately one quarter of *Pst*-sRNA loci did not overlap with any known functional annotation (Table 2). These included both microRNA-like loci. Eighteen loci overlapped with fungal repeat elements from RepBase, as predicted by RepeatMasker. These included DNA transposons of the hAT, MuDR, and *Hop* families. About half of all *Pst*-sRNA loci overlapped with tRNA genes predicted by tRNAScan-SE [48]. The mean and mode read length mapping to tRNA genes was 31 nt. Consequently, tRNA-derived fragments were a major source of the longer sRNAs visible in the overall length distribution (Fig. 3). Many tRNA-derived sRNA loci were characterized by two groups of reads mapping to the 5′ and 3′ boundaries of predicted tRNA genes. tRNA-derived fragments (tRFs) were described in small RNA libraries from the rice blast fungus *Magnaporthe oryzae* [25]. However, the tRFs in our libraries varied widely by length, start position, and stop position, and did not appear specifically processed.

**Fig. 6:**
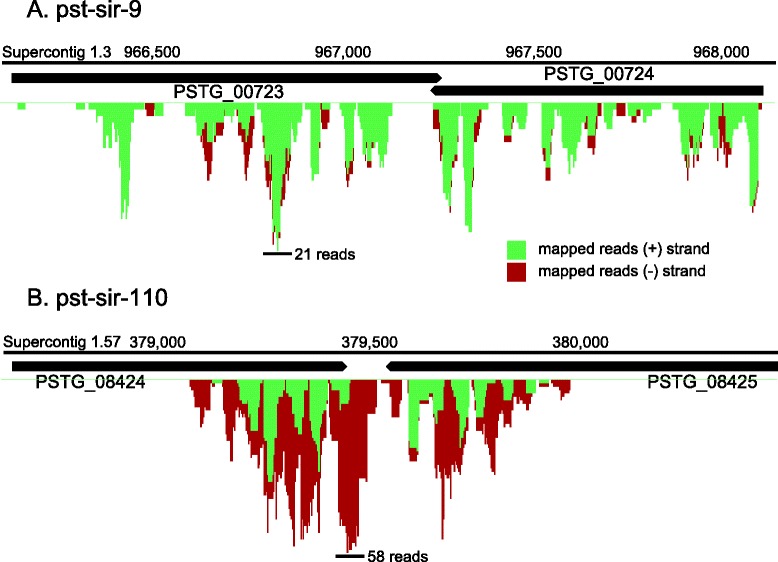
Inverted repeat-associated *Pst-*sRNA loci. Distribution of mapped reads for two gene-associated sRNA loci. **a**. pst-sir-9. **b**. pst-sir-110. Transcripts from each tail-to-tail gene pair have long regions of near-perfect complementarity. Bars indicate the peak number of overlapping reads (depth)

Several thousand *Pst*-sRNA reads from 33 loci mapped within or near predicted genes (Table 2). Upon scrutinizing these genomic regions, it was discovered that 14 of these loci were long inverted repeat regions, sometimes with borders extending well past the predicted 3′ end of the genes they overlapped. Of particular interest were loci with hundreds of sRNA reads mapping to a pair of genes in a tail-to-tail arrangement (Fig. 6). Reads mapping to one gene in a pair were complementary to a corresponding region of the other gene. In fact, some sequences had two perfect genomic matches, one on each DNA strand on opposing sides of the inverted repeat, making their true mapping location ambiguous. Read lengths at these loci varied from 18–22 nt with a mode of 21 nt. Reads were unevenly dispersed across the loci and showed no evidence of precise miRNA-like processing.

The two genes in the pst-sir-9 locus are closely related, with high sequence homology throughout the coding region (Fig. 6a). In contrast, the genes in the pst-sir-110 locus have unrelated coding sequences, yet the 3′ end of the coding region of *PSTG_08424* is similar in sequence to the predicted 3′ UTR of *PSTG_08425*. All four genes featured in Fig. 6 have ESTs indicating their expression [38]. This arrangement coupled with sRNA production is reminiscent of *cis-*natural antisense transcription (*cis-*NAT) described previously in fungi [49]. However, these genes are not actually antisense pairs, but rather close homologs. If transcription of one gene were to continue past the normal termination site and into the neighboring gene, the result might be a long hairpin-shaped transcript that is capable of generating sRNAs that target one or both genes. More investigation of transcript abundance and variants is needed to determine the biological significance of these gene pairs.

### Small RNA target prediction

If *P. striiformis* employs small RNA to regulate endogenous fungal gene expression, then the sRNA sequences described in this study will share regions of complementarity with protein-coding sequences. Likewise, recent discoveries in *Botrytis* [26] provided evidence that fungal sRNAs can enhance virulence by disrupting host genes. We used software programs to predict a list of sRNA-target pairs in the gene sequences of both *P. striiformis* and *T. aestivum*.

In general, target prediction programs first align a given sRNA sequence to more or less complementary regions in a database of target transcripts. Likelihood scores are calculated via criteria from empirically-validated sRNA-target pairs, or by predicting the binding affinity of the sRNA, given the native secondary structure of the target. If the score meets a user-defined cutoff, then the program outputs the sRNA sequence paired with its predicted target gene accession. To date, no software has been designed specifically to predict small RNA targets in fungi. Therefore, three different target prediction tools were run and compared: psRNATarget [50], TAPIR FASTA [51], and TargetFinder [52]. All three programs have been used on a wide range of species, and were featured in a comparative study to determine score cutoffs that optimize precision and recall in both *Arabidopsis* and non-model plants [53].

**Fig. 7:**
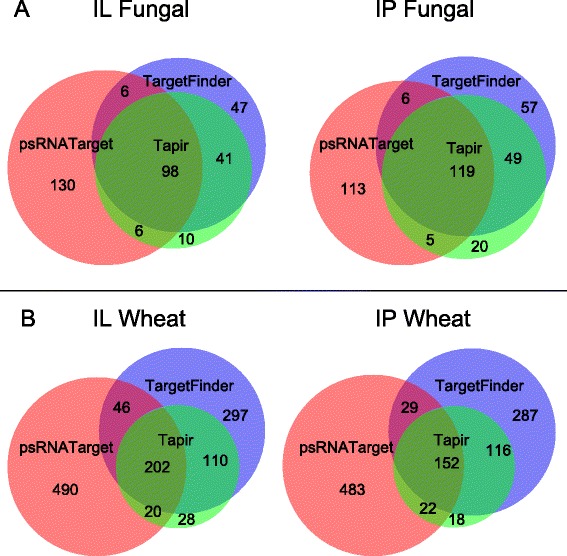
Target prediction program comparison. Venn diagrams comparing predicted targets of *Pst-*sRNA in stripe rust and wheat, according to three software programs. **a**: sRNA-target pairs from Infected Louise (left) and Infected Penawawa (right) in PST-78 gene sequences. **b**: sRNA-target pairs from Infected Louise (left) and Infected Penawawa (right) in the Washington Wheat Transcriptome

We selected *Pst*-sRNA sequences that were 20–30 nt in length and with at least one read in every replicate of IL and/or IP. This equalized inputs to the three programs (psRNATarget discards sRNA sequences >30 nt in length), and avoided spending computing resources on the least-abundant *Pst*-sRNAs. TargetFinder, TAPIR, and psRNATarget were used to predict targets in both *Pst* and wheat transcripts. The sRNA-target pairs output by each program were counted and compared (Fig. 7). Approximately one third of *Pst-*sRNA sequences were predicted to target more than one gene. The output from TAPIR fit almost entirely inside the output from TargetFinder (Fig. 7a). In contrast, a substantial fraction of psRNATarget’s output was unique to that program, and not shared by the other two. There were more total sRNA-target pairs predicted in wheat than in *P. striiformis* (Fig. 7b). This probably reflects the plant-oriented design of the programs, and the fact that the target transcriptome for wheat (1.3x10^8^ bp) is five times larger than for *Pst* (2.3x10^7^ bp). The overlapping regions of Fig. 7 contain sRNA-target pairs predicted by two or more programs.

### Target genes in *P. striiformis*

Target genes that were predicted by two or more programs were screened to determine interesting candidates for functional analysis. Predicted fungal target genes were searched via the BROAD Institute’s *Puccinia* Group database [38], and were also BLASTed against the nonredundant protein database from NCBI. BLAST results were analyzed with InterProScan and then mapped to gene ontology (GO) terms using Blast2GO software.

Out of 78 target genes with GO terms, 10 genes (13 %) were assigned the molecular function “kinase activity” (GO: 0016301). This term was found with greater frequency in the target list than in the *Pst* genome overall (*p* = 0.003, Fisher’s Exact Test). The kinase genes included members of the FunK1 family of fungal protein kinases, an atypical phosphatidyl inositol 3′ kinase-related protein kinase (PIKK), and an ULK/ULK serine/threonine protein kinase. Genes related to vesicle-mediated intracellular transport, G protein signaling, and protein turnover were also prevalent (Table 3). The abundance of genes related to protein phosphorylation and signal transduction suggests a role for *Pst*-sRNAs in regulating mycelium development during early infection. A transposase gene was also a predicted target, implying that small RNAs may silence transposons, as in many other organisms.

**Table 3:** Predicted targets of *Pst*-sRNAs in stripe rust

sRNA	Target gene	Description	BLAST hit species	E-Val
IP20_38	PSTG_16713	choline kinase	*Puccinia graminis*	0.00E + 00
IL20_14	PSTG_09361	E3 ubiquitin ligase	*Puccinia graminis*	0.00E + 00
IP20_34	PSTG_14619	chitin synthase	*Trametes versicolor*	1.25E-35
IP20_35	PSTG_04555	FunK1 4 kinase	*Puccinia graminis*	0.00E + 00
IL21_27	PSTG_02370	malate synthase	*Puccinia graminis*	0.00E + 00
IL21_50	PSTG_14325	mutator-like transposase	*Puccinia graminis*	3.80E-82
IP21_25	PSTG_00041	peroxisomal targeting signal receptor	*Puccinia graminis*	0.00E + 00
IP22_01	PSTG_08072	Atypical PIKK protein kinase	*Puccinia graminis*	1.50E-37
IP22_11	PSTG_17566	protease Ulp2-like	*Puccinia graminis*	0.00E + 00
IP24_09	PSTG_08907	regulator of G protein signaling	*Puccinia graminis*	0.00E + 00
IL20_45	PSTG_02623	Rho-GEF-containing protein	*Melampsora larici-populina*	8.30E-132
IP21_37	PSTG_04673	septin	*Puccinia graminis*	0.00E + 00
IP20_49	PSTG_07126	sorting nexin mvp1	*Rhodosporidium toruloides*	7.89E-66
IL20_15	PSTG_10387	transport protein Avl9	*Puccinia graminis*	0.00E + 00
IL20_24	PSTG_00809	trapp complex subunit Trs31	*Puccinia graminis*	5.40E-147
IP21_45	PSTG_14191	type II pantothenate kinase	*Puccinia graminis*	0.00E + 00
IL21_04	PSTG_02562	u3 snoRNA-associated protein 6	*Puccinia graminis*	0.00E + 00
IP24_11	PSTG_04500	ULK ULK protein kinase	*Puccinia graminis*	0.00E + 00

Effector protein expression is an important step in the development of biotrophic pathogens. Prediction of effectors has focused on small, cysteine rich proteins that contain a secretion signal and a known effector motif or nuclear localization signal [54]. In our data, 22 fungal target genes encode proteins with a predicted signal peptide cleavage site according to SignalP 4.1 [55]. Of these, 14 were less than 120 amino acids in length with at least six cysteine residues each. Many of these genes shared an N-terminal [Y/F/W]xC motif, which is common among effector candidates in haustoria-forming fungi [56]. Cantu and colleagues (2013) organized the predicted secretome into protein tribes, based on genomic re-sequencing and RNA-seq of several stripe rust isolates [10]. BLAST searches of that data set revealed that the genes in our target list matched genes in Tribe 3 and Tribe 411; members of these tribes were found to be expressed in haustoria and infected tissue. Furthermore, the gene expression of secreted proteins varies during early, middle, and late infection, raising the possibility of strategic regulation of effectors over time [10]. These effector candidates will be among the first genes tested for functional analysis of specific sRNA-target pairs.

### Target genes in wheat

As with the fungal targets, wheat target genes predicted by at least two out of three programs were annotated using Blast2GO software. BLAST hits were compiled for 429 genes that matched sequences in related species or wheat progenitors; 359 of these were assigned at least one GO term. The top molecular function terms assigned were ATP binding (59 genes, GO:0005524), zinc ion binding (24 genes, GO:0008270), and DNA binding (23 genes, GO:0003677). Interestingly, BLAST searches of the wheat target list revealed many genes carrying the features of known resistance genes (Table 4). NBS-LRR proteins and serine/threonine kinases are known to contribute to rust resistance in cereals [57]. Other targets matched proteins involved in programmed cell death and senescence. If fungal small RNAs cross the extrahaustorial space and enter plant cells, then the host’s own silencing machinery could be used to block components of the plant disease response [26]. Going forward, sRNA-target pairs will be combined with gene expression data to search for defense-related genes that are unexpectedly downregulated in response to infection, possibly implicating a small RNA-based effector.

**Table 4:** Predicted targets of *Pst*-sRNAs in wheat

sRNA	Target Gene	Description	BLAST Hit Species	E-Val
IL21_18	JP849598.1	ABC transporter C family member 5	*Aegilops tauschii*	0.0E + 00
IP21_89	JP868961.1	barley stem rust resistance protein Rpg1	*Hordeum vulgare*	0.0E + 00
IP20_54	JP844160.1	chitin-inducible gibberellin-responsive protein	*Brachypodium distachyon*	0.0E + 00
IP20_21	JP860791.1	cysteine-rich receptor-like protein kinase 26	*Aegilops tauschii*	0.0E + 00
IL20_15	JP878991.1	death-inducer obliterator 1-like	*Brachypodium distachyon*	0.0E + 00
IP21_89	JP914868.1	NBS-LRR protein RGA2-like	*Aegilops tauschii*	0.0E + 00
IL21_27	JP903853.1	NBS-LRR protein RPM1-like	*Triticum urartu*	0.0E + 00
IL20_45	JP921169.1	NBS-LRR protein RPP13-like	*Oryza brachyantha*	4.3E-69
IL21_31	JP921571.1	NBS-LRR protein Rps2-like	*Triticum urartu*	0.0E + 00
IL21_46	JP940321.1	endoribonuclease dicer homolog 2a-like	*Brachypodium distachyon*	1.3E-102
IL21_52	JP939823.1	G-type lectin S-receptor-like S/T kinase	*Brachypodium distachyon*	6.5E-109
IP20_47	JP884254.1	LRR receptor-like kinase erecta	*Triticum aestivum*	7.8E-51
IL21_69	JP871726.1	MYB transcription factor	*Zea mays*	6.6E-69
IL22_29	JP875481.1	NBS-LRR protein	*Aegilops tauschii*	0.0E + 00
IL20_26	JP930830.1	NBS-LRR protein RGA4-like	*Triticum urartu*	1.1E-128
IL21_52	TC371700	receptor-like protein kinase	*Triticum urartu*	0.0E + 00
IP24_09	JP867166.1	receptor-like protein kinase feronia	*Aegilops tauschii*	0.0E + 00
IP25_06	JP821112.1	senescence-associated protein	*Medicago truncatula*	9.7E-41
IL21_82	JP828505.1	serine threonine protein kinase EDR1 isoform	*Hordeum vulgare*	0.0E + 00
IP21_53	JP927012.1	serine threonine protein kinase PBS1	*Aegilops tauschii*	1.1E-30
IL21_05	TC432374	wall-associated receptor kinase 5-like	*Aegilops tauschii*	1.7E-92
IP24_09	JP871864.1	WD repeat-containing protein 74	*Triticum urartu*	9.8E-105

## Conclusion

This research contributes an inventory of small RNAs from one member of an important group of plant pathogens: the rust fungi. The expression of RNAi genes in *P. striiformis* led to the hypothesis that this organism possesses functional small RNAs. By obtaining a broad sample of small RNA from infected wheat, hundreds of novel sRNA sequences from *Pst* were identified. The recalcitrance of this pathogen to axenic culture provided both the challenge and the advantage of using infected plant tissue to construct sequencing libraries. Unlike many studies of plant-pathogenic fungi, which have used lab-cultured tissue, the sRNAs discovered in this study are certainly present during early stripe rust infection, and cannot be artifacts of growth on sterile media. The tradeoff, of course, is the possibility that some sequences attributed to the fungus actually originate in wheat. However, given the filtering method used herein, such contaminating sequences would have to map perfectly to the *Pst* genome, be transcribed exclusively during infection, and have never been observed in previous wheat transcriptome or miRNA studies. We are confident that this pipeline accurately identified legitimate fungal sequences, though perhaps at the expense of losing some reads from noncoding RNA families that are conserved between the two organisms. Small RNA libraries from purified haustoria or germinated urediospores might increase the relative proportion of fungal-specific reads, but would miss the diversity of sRNA sequences found in the complete infectious mycelium.

The size distribution, position-specific nucleotide preferences, and accumulation of specific sequences all suggest that *P. striiformis* possesses an endogenous sRNA biogenesis pathway. Rather than an arbitrary mix of degradation products, *Pst*-sRNAs share many characteristics with small RNAs identified in other RNAi-equipped organisms. Most *Pst*-sRNAs are produced from distinct genomic locations that give rise to large numbers of sequences with similar or identical lengths. Some of these loci are structurally analogous to microRNA loci, while others come from genes, inverted repeats, and transposons. We conclude that the sRNAs identified in this study are far more similar to those from RNAi-equipped fungi than from RNAi-deficient species. To assess the impact of *Pst-*sRNA in gene regulation, the next step will be to combine these findings with transcriptome data, including both intact and cleaved mRNAs. Specific candidate sRNA-target pairs can be tested via a modified 5′ RACE assay to detect transcript slicing at sites that correspond to sRNA sequences [58]. Site-specific cleavage, if detected, will support the predictions made by this study, and provide the empirical framework for developing the first fungal-oriented target prediction software.

Multiple software programs predicted that endogenous *Pst-*sRNAs may target fungal and/or wheat genes for post-transcriptional silencing. On the fungal side, the number of target genes involved in protein phosphorylation suggest that development-related signaling pathways may be regulated in this manner. Furthermore, more than a dozen target genes code for small, secreted cysteine-rich proteins that are currently considered effector candidates. The mechanism by which fungi rapidly gain and lose virulence/avirulence genes is a major area of plant pathology research [8]. Rather than lose avirulence proteins outright via mutation, pathogens might instead use sRNAs to silence genes that would otherwise elicit an immune response. Regarding effector candidates, it was recently observed that there are surprisingly few presence/absence polymorphisms in the genomes of stripe rust isolates with very different virulence profiles [10]. One current hypothesis is that differential virulence is caused by allelic variation at the protein level. Nonetheless, it is also plausible that even a synonymous mutation at the mRNA level might create or disrupt an sRNA binding site, thereby altering expression levels and leading to the same differential virulence. Differential epigenetic control of effector alleles via noncoding RNAs is yet another possibility [59]. As more effector genes are predicted in *Puccinia spp.*, the nucleotide sequences of such genes should be checked for potential sRNA target sites.

On the plant targeting side, numerous genes bearing leucine-rich repeats and other hallmarks of resistance genes make attractive targets for functional analysis. The aforementioned 5′ RACE assay may be used, as well as its high-throughput counterpart, degradome sequencing [60, 61]. An sRNA and its target may also be transformed into a more tractable genetic system, such as *Nicotiana benthamiana*, to test whether PTGS occurs *in vivo*.

We did not observe convincing evidence that production of fungal sRNA varies depending on the cultivar of infected wheat. The specific *Pst-*sRNA sequences and their expression levels collected from the susceptible wheat cultivar ‘Penawawa’ were very similar to those from the HTAP-resistant cultivar ‘Louise’. The failure to detect significant differences may reflect a lack of plasticity in the fungal response to plant defenses. Or, as a histological study of stripe rust development found, hyphal growth on a resistant cultivar matched and even exceeded the growth rate on a susceptible cultivar during the first few days of infection [62]. Thus, our tissue collection at 4 days post inoculation may have missed the full induction of plant defenses and corresponding stress responses in the pathogen. A time course study that includes sRNA collection from later infection could shed light on this question.

The results of this study are consistent with the current proposed model of host-induced gene silencing. Silencing signals from the plant, whether taken up by the fungus as antisense precursors or mature sRNA fragments, may operate via the fungus’s own RNAi machinery. Although HIGS experiments to date have been engineered via transient transformation, it is entirely possible that plant-endogenous cases of HIGS exist. The small RNA libraries developed in this study can be used to investigate both sides of a potential interspecies RNA exchange.

## Methods

### Plant varieties and growth conditions

Wheat seeds of the varieties ‘Louise’ and ‘Penawawa’ were germinated on wet filter paper for two days, then planted in one-gallon soil-filled pots, one seedling per pot. Pots were kept in a climate-controlled chamber with 16 h light at 25 °C; 8 h dark at 15 °C. Plants were inoculated at 6 weeks (42 days) after planting, when expanded flag leaves showed visible ligules, but before heading (Feekes Growth Stage 9).

### Inoculation and tissue harvest

A sample of the isolate PSTv37 (PST-100) was obtained courtesy of Dr. Xianming Chen (USDA-ARS, Pullman, WA). Urediniospores were increased on Penawawa seedlings prior to the experiment. Spores were stored at 4 °CC with calcium sulfate desiccant until just before use. Spores were diluted by a factor of 10 (w:w) with talcum powder. This mixture was applied liberally to both sides of flag leaves using gloved fingers. Half of the plants in each variety were spore-inoculated; the other half were mock-inoculated with pure talcum powder and subjected to identical conditions. Three biological replicates (individual plants) were inoculated in each treatment group.

After inoculation, plants were misted lightly with distilled water. Plastic sleeves were placed around the mock-inoculated pots to prevent contamination. Plants were placed in a sealed dew chamber at 10 °C with 95 % relative humidity. After 24 h, they were removed from the dew chamber and placed in a climate-controlled chamber for an additional 3 days (16 h light at 16 °C; 8 h dark at 8 °C), totaling 4 days post-inoculation. Whole flag leaves were harvested just above the ligule with scissors and placed in a sealed 15 mL Falcon tube, then immediately frozen in liquid N_2_.

### RNA extraction and library construction

Frozen tissue was ground in liquid nitrogen using a mortar and pestle. After grinding, each sample was divided, and two parallel RNA extractions were performed: one for total RNA, and the other for the small RNA fraction only (≤200 nt). The mirVana RNA isolation kit (Life Technologies, Thermo Fisher, USA) was used for both extractions. RNA was quantified with a NanoDrop 1000 (Thermo Fisher, USA) and with a Bioanalyzer 2100 (Agilent, USA) to check RNA integrity. The sRNA fraction was used for cDNA library preparation using the Ion Torrent Total RNA-seq Kit Version 2 (Life Technologies, USA). Barcoded sequencing adapters enabled multiplexed sequencing of all 12 sample libraries. High-throughput sequencing was performed using the Ion Proton platform (Life Technologies, USA) at the WSU Molecular Biology and Genomics Core.

### RT-PCR for fungal RNAi genes

Total RNA from infected and uninfected Penawawa leaves was treated with DNase (New England BioLabs, USA) and reverse transcribed using SuperScript III (Invitrogen, USA). PCR was performed using AmpliTaq Gold polymerase (Life Technologies, USA). Samples were pre-heated for 8 min at 95 °C, followed by 35 cycles of PCR with the following conditions: 15 s at 95 °C; 30 s at 52 °C; 60 s at 72 °C. Wheat GAPDH and *P. striiformis* actin were used as controls. Fungal-specific primer pairs were designed with NCBI Primer BLAST to avoid amplification of wheat genes (Table 5). PCR products were visualized on a 1 % agarose gel containing TAE buffer and ethidium bromide. Bands of the target lengths were excised from the gel, and DNA was extracted using the QIAquick Gel Extraction Kit (QIAGEN, Netherlands). Sanger sequencing was performed at Elim BioPharm (USA).

**Table 5:** Primer sequences used in RT-PCR experiments

Primer name	Sequence (5′ to 3′)	Expected product size
PSTG_06326 F	CACTCAGTGTTTTGCCGTGG	800
PSTG_ 06326 R	AGTATGCCGGTGTAGCGATG	
PSTG_15713 F	GAAGCGTGCAGAGTATTGCG	887
PSTG_15713 R	CAAAGTGTGCGCGGGATATG	
PSTG_15184 F	GGGTCGAAATCCGCGATAGT	294
PSTG_15184 R	TGGCCGTCCGGTTTAGAATC	
PSTG_03098 F	TAGCACCAACTGATGCCAGG	273
PSTG_03098 R	AACCTCTTCCAGATGCAGCC	
PST_actin F	CTATTGACTGAAGCTCCTCTCAATCCCA	213
PST_actin R	CGCATGAGGTAGAGCGTAACCTT	
TAE_GAPDH F	CAACGCTAGCTGCACCACTA	161
TAE_GAPDH R	TTCCACCTCTCCAGTCCTTG	
IP_2101 F	TTCCCAGGAAGCGATGAGC	120
IP_2102 F	AGCAGCAACCCTGTCGGC	120
IP_2103 F	TTGCAAGGACTCCGGAGAGGC	120
IP_2105 F	TTTAGCGAGTTGGAGTGAT	120
IP_2106 F	TAGGGACCTCGATAGGACA	120
ta-miR_2002/2075 F	TGAGATGAGATTACCCCAT	120
U6 snRNA F	CCGATAAAATTGGAACGATAC	195
RTQ-universal R	CGAATTCTAGAGCTCGAGGCAGG	—
miRTQ	CGAATTCTAGAGCTCGAGGCAGGCGACATGGCTGGCTAGTTAAGCTTGGTACCGAGCTCGGATCCACTAGTCCTTTTTTTTTTTTTTTTTTTTTTTTVN	—

### Bioinformatics pipeline

Ion Torrent software (Life Technologies, USA) was used to trim adapter and barcode sequences, assign reads to each library based on barcode, and filter out low-quality reads (average PHRED <15). Mapping of 18–40 nt reads was performed using Butter 0.3.2, a variant of Bowtie optimized for small RNA and included in the ShortStack package [44]. Butter iteratively places reads, such that reads with multiple possible alignments are mapped to a single location in the resulting BAM file. Only perfect matches to the *P. striiformis* PST-78 draft genome were accepted. BAM mapping files from Butter were imported into CLC Genomics Workbench 7 (QIAGEN, Netherlands), where sequences were tabulated and counted using the small RNA analysis toolkit. Mapped sequences that were present in both infected and uninfected libraries were removed from the infected libraries to enrich the library for fungal sequences. The resulting sequence list was mapped to the Washington Wheat Transcriptome [40]. Small RNA reads that mapped to the coding (+) strand with zero mismatches were discarded. The process was repeated using MiRBase Release 21, which has 116 miRNA precursors for *Triticum aestivum*. Size distribution and 5′ nucleotide bias were performed in CLC Genomics Workbench 7. Empirical Analysis of Differential Gene Expression was also performed in CLC, using the edgeR method described in [63].

### RT-PCR for *Pst-*sRNA sequences

A portion of the original size-selected sRNA extract was used to validate RNA-seq results via endpoint RT-PCR, as described in [41]. Small RNAs were polyadenylated with Poly(A) polymerase (NEB, USA) and then reverse-transcribed with a specialized long RT primer. The target product size, including the sRNA sequence and RT primer sequence, was ~120 bp in length. Products were amplified using an sRNA-specific forward primer and a universal reverse primer. Samples were pre-heated for 8 min at 95 °C, followed by 40 cycles of PCR with a combined annealing and extension step: 15 s at 95 °C; 60 s at 62 °C. All primer sequences are found in Table 5.

### Discovery of miRNA-like loci

The ShortStack package was obtained from the Axtell Lab (http://github.com/MikeAxtell/ShortStack) and installed on a Linux workstation running Perl 5.14. Trimmed sRNA reads and the PST-78 genome were input into ShortStack; the program was run using the following options: –mismatches 0, –mindepth 20, –pad 200, –dicermin 18, –dicermax 35, –miRType ‘plant’, –phasesize ‘all’. Resulting GFF3 annotation files, carrying the genomic coordinates of ShortStack-determined sRNA loci, were imported into CLC Genomics Workbench as tracks on the genome. Maple (miRNA discovery) was run using default settings. Scores generated by Maple fall between 0 (poor) and 1 (excellent), plus an overall verdict (PASS/FAIL) for each putative miRNA cluster. Loci receiving a PASS verdict were automatically output to RNAfold to graphically display secondary structure (http://www.tbi.univie.ac.at/).

### Overlap of *Pst-*sRNA loci with genome annotations

Repeat elements specific to fungi (2,350 loci) were downloaded from RepBase 20.01 (http://www.girinst.org/repbase/). RepeatMasker 4.0.5 was run on the stripe rust genome using the following options: −nolow, −no_is, −gff. Next, tRNAScan-SE 1.3.1 was run on the whole genome sequence using default parameters. A Perl script was used to convert the output of tRNAScan-SE to GFF. Current Rfam and gene annotations were downloaded from the Broad Institute *Puccinia* group as GTF files (http://www.broadinstitute.org/annotation/genome/puccinia_group/MultiHome.html). Annotation files were imported into CLC Genomics Workbench 7. A track list was constructed over the PST-78 genomic sequence that included ShortStack loci and all annotations mentioned above. Then, the tool “Annotate with Overlap Information” was used to find the number of ShortStack loci with boundaries that overlapped each annotation feature (genes, repeats, tRNAs, etc.). The tool “Extract Reads Based on Overlap” was used to obtain the RNA-seq reads corresponding to each annotation feature.

### Target prediction

*P. striiformis* gene sequences were downloaded from the Broad Institute in FASTA format [38]. Wheat sequences were downloaded from the Washington Wheat Transcriptome database [40]. TargetFinder 1.6 is a Perl program obtained from the Carrington Lab (https://github.com/carringtonlab/TargetFinder). By default, TargetFinder searches a single sRNA sequence against a target gene database. A Perl script was written to loop TargetFinder for a list of many sRNA sequences, and output the results as comma-separated text. TargetFinder was run using default settings and a score cutoff ≤ 3.5. The psRNATarget program is available as a browser-based tool (http://plantgrn.noble.org/psRNATarget/). Default settings were used with a score cutoff ≤ 2.5. TAPIR 1.2 is a Perl program obtained from the Van de Peer lab (http://bioinformatics.psb.ugent.be/webtools/tapir/). TAPIR was run in FASTA mode using default settings and a score cutoff ≤ 3.5. Output from each program was limited to 25 hits for each small RNA.

Output from all three programs was manipulated into the text format “sRNA_accession;TargetGene_accession\n” to create comparable lists of sRNA-target pairs. Lists were compared using the browser-based BioVenn tool and visualized as area-proportional Venn diagrams [64].

### Gene ontology of predicted targets

Small RNA-target pairs predicted by two or more software programs were extracted from BioVenn output; FASTA files with the nucleotide sequences of these target genes were imported into Blast2GO 3.0 (BioBam, Spain). BLASTx was run on fungal target gene sequences against the nonredundant (NR) protein database (subset fungi, taxid: 4751) at NCBI. InterProScan 5.9 was run on BLAST results, and GO terms were assigned to target gene sequences. This process was repeated for the entire list of predicted PST-78 genes [38]. The number of genes with the GO term “kinase activity” (GO: 0016301) in the target list was compared with the total gene list using Fisher’s Exact Test in R (version 3.1.1). Predicted effector proteins in the *P. striiformis* genome were downloaded from supplemental files in [10]. Similarly, the list of wheat genes targeted by *Pst-*sRNAs was BLASTed against the NR protein database (subset viridiplantae, taxid 33090).

### Graphic design

Figures 5, 6, 7, and Additional file 1 were made using InkScape (www.inkscape.org).

## Availability of supporting data

The data set supporting the results of this article is available in the NCBI Sequence Read Archive, accession SRP060546, BioProject PRJNA289147. http://trace.ncbi.nlm.nih.gov/Traces/sra/sra.cgi?study=SRP060546

## Additional files

Additional file 1: 
**Bioinformatics Pipeline.** Graphical summary of the bioinformatic pipeline used to obtain putative *Puccinia striiformis* small RNAs (*Pst-*sRNAs). The total sRNA library consists of mostly wheat reads (green) with a small fraction of stripe rust reads. After mapping to the stripe rust genome, discarding reads present in uninfected controls, and discarding sequences matching wheat miRNA and protein-coding sequences, the library is increasingly enriched for stripe rust reads (orange). Sequences discarded at each step are shown as arrows to the right. (PDF 36 kb) Additional file 2: 
**Top 100** ***Pst*** **-sRNA sequences sorted by abundance.** Each Pst-sRNA was uniquely named based on the treatment in which it was found, length, and rank abundance in that treatment. Fungal reads in sRNA libraries from two infected cultivars. IP = Infected Penawawa; IL = Infected Louise. Counts represent the sum of three replicates. (CSV 5 kb)

## Abbreviations

PTGS: Post-transcriptional gene silencing
sRNA: small RNA
miRNA: microRNA
siRNA: small interfering RNA
HTAP resistance: High-temperature adult-plant resistance
FDR: False discovery rate
GO: Gene ontology
