# Supplementary figures and images for: Small RNAs from the wheat stripe rust fungus (Puccinia striiformis f.sp. tritici)
Source: BMC Genomics. 2015 Sep 21;16(1):718. doi: 10.1186/s12864-015-1895-4 (PMC4578785; doi:10.1186/s12864-015-1895-4)

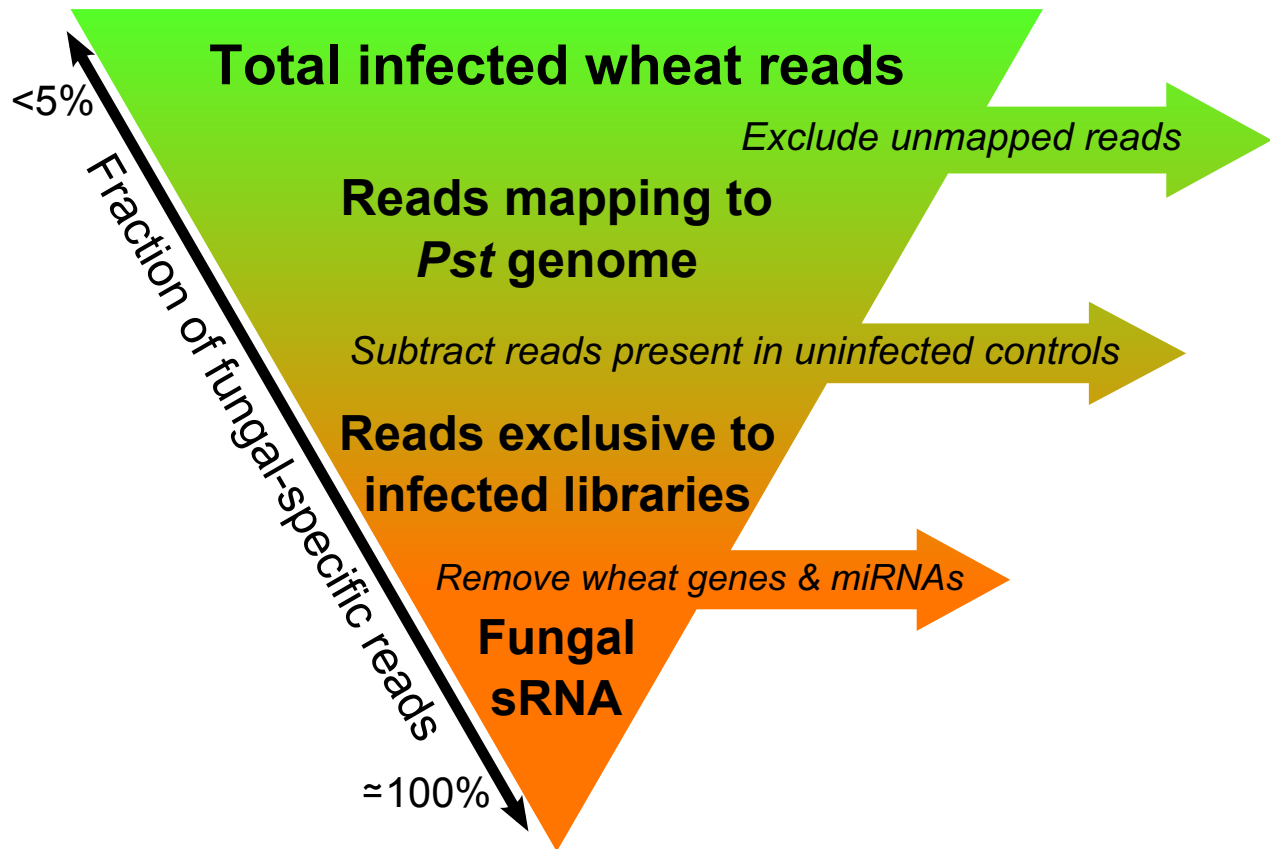

Supplement: Additional file 1: — Bioinformatics Pipeline. Graphical summary of the bioinformatic pipeline used to obtain putative Puccinia striiformis small RNAs (Pst-sRNAs). The total sRNA library consists of mostly wheat reads (green) with a small fraction of stripe rust reads. After mapping to the stripe rust genome, discarding reads present in uninfected controls, and discarding sequences matching wheat miRNA and protein-coding sequences, the library is increasingly enriched for stripe rust reads (orange). Sequences discarded at each step are shown as arrows to the right. (PDF 36 kb) [file 12864_2015_1895_MOESM1_ESM.pdf]
